# Schober Test and Its Modifications Revisited—What Are We Actually Measuring? Computerized Tomography-Based Analysis

**DOI:** 10.3390/jcm11236895

**Published:** 2022-11-22

**Authors:** Oded Hershkovich, Michael Paul Grevitt, Raphael Lotan

**Affiliations:** 1 Department of Orthopedic Surgery, Wolfson Medical Center, Holon 5822012, Israel; 2 Sackler School of Medicine, Tel Aviv University, Tel Aviv 6997801, Israel; 3 Centre for Spinal Studies and Surgery, Queen’s Medical Centre, Nottingham NG7 2UH, UK

**Keywords:** Schober test, range of motion, lumbar, modified

## Abstract

Objective: Examine Schober test’s (ST), Modified ST (MST), and Modified–Modified ST (MMST) surface markers’ accuracy in spanning lumbar L1-S1 motion segments and repeatability related to actual patient anatomy as measured on sagittal CT scans. Methods: The study included 25 patients of varying heights, weights, and gender without prior spinal surgery or deformity. Researchers assessed patients’ CT scans for ST, MST, and MMST skin levels of the measured cephalic and caudal endpoints. Results: The original ST failed to include at least one lumbar motion segment in all patients, omitting the L1-L2 motion segment in 17 patients and the L2-L3 in another eight. The additional cephalic length of the MST did not improve the inclusion of the actual L1-S1 components. The MMST measured 19 ‘patients’ entire L1-S1 motion segments, reaching a 76% accuracy rate. WMST, measuring 16 cm (instead of MMST’s 15 cm), improved the measurement significantly, measuring the L1-S1 motion segments in all cases (with 100% accuracy). Conclusion: ST and its modifications fail to span the L1-S1 motion segments and are thus prone to underestimating lumbar spine motion. This study shows that the WMST is much more accurate than previous modifications and is a better tool for evaluating lumbar spine motion.

## 1. Introduction

Low back pain (LBP) management is challenging. When examining the International Classification of Function, Disability, and Health [1], LBP disability involves dysfunction through impairment, activity limitation, and participation restriction. Physicians must recognize all three dysfunction components in the evaluation and treatment to prevent disability [2]. Range of motion (ROM) is a significant impairment factor to be addressed in the follow-up and monitoring of LBP and the result of therapy [3,4,5]. Lumbar ROM measurements on their own are not an accurate predictive tool for actual patient status [6,7,8]. They should not be used alone when aiming to quantify disability [6]. However, ROM estimation remains a vital part of detecting impairment and is integral to treatment planning [5,9,10]. Lumbar ROM is a crucial parameter in determining disability in the context of worker’s compensation, insurance claims and litigation worldwide [2,3]. Lumbar ROM assessment methods should be investigated to ensure accuracy, reliability, and repeatability.

Nevertheless, the current literature describes only a few lumbar ROM measurement methods or devices with good reproducibility properties [11]. Some of the instruments available include the Draughtsman’s flexible ruler [12,13,14,15], finger-floor method [16], double inclinometer method [17,18,19,20], tape measurement method [21,22,23,24,25,26,27], and devices such as the Cybex EDI-320 (Electronic Digital Inclinometer) [28,29,30] and the Back Range of Motion device (BROM II) [31]. Good reproducibility properties were reported with the tape measurement and the Double Inclinometer (DI) methods for measuring lumbar flexion ROM. However, the clinical use of DI is unpopular, as it is time-consuming and requires skill and training [20,21]. The tape measurement method is commonly used to measure active lumbar ROM. The method requires no special equipment, skill, or training in its use.

The original Schober test uses a tape held over the spine between the lumbosacral junction and 10 cm above it [24]. The challenge in precise localization of the lumbosacral junction led to an adaptation to the original test [25] by marking a point 5 cm below and 10 cm above the lumbosacral junction. When published, the Modified Schober Test (MST) was compared to L1-S1 radiographic measurements on a small group of patients and found a very high correlation. Further studies reported high accuracy and reproducibility [23,26,27]. Nonetheless, the use of the MST has been challenged in the last decade, with others reporting lower accuracy rates [22,32]. In one study, fifty healthy subjects were evaluated for lumbar flexion, and the authors stated that only four spinal segments (L2-S1) were actually included in the MST measurement of lumbar spine flexion [32]. Based on these findings, the utility of this method was questioned on both scientific and clinical grounds and echoed earlier criticism concerning the distance between skin landmarks [22]. These authors used different distances between the posterosuperior iliac spine (PSIS) and a midline landmark at 5, 10, 15, and 20 cm cephalad. They concluded that the 15 and 20 cm segments above the PSIS’s midline contributed little to an overall measure of lumbar flexion. Based on these results, they proposed a second adaptation of the original Schober test: The Modified–Modified Schober Test (MMST) using the 15 cm distance cranial to PSIS landmarks.

Only a few studies confirmed MMST validity. In one of those, 15 patients with chronic LBP had MMST measurements by three experienced physical therapists [21]. Pearson correlation coefficients for test–retest reliability varied from 0.78 to 0.89 for lumbar flexion, and intra-class correlation coefficients (ICC) for inter-rater reliability were 0.72. In another study, the authors concluded that intra-examiner reliability was good, but inter-examiner reliability was low [33].

To summarize, as ROM is a substantial measure of physical limitation and improvement, and is frequently used as a treatment goal by the clinician, measurement methods with good psychometric properties and ease of use are needed. The Schober test is used to diagnose Ankylosing spondylitis and other spondyloarthropathies, assess disability in the clinical setting and litigation and assess treatment results. These tests are widely used by orthopedic surgeons, rheumatologists, rehabilitation specialists and physiotherapists. The tape measurement method remains the primary option for the clinician; however, all the suggested modifications lack accuracy. The MMST, the last modification of the Schober test, includes two improvements: (1) the use of the PSIS as opposed to the lumbosacral junction to include the L5-S1 movement while eliminating the difficulty of finding the lumbosacral junction: and (2) a 15 cm cephalad landmark to include all lumbar motion segments. However, the reproducibility properties of the MMST need to be better established.

This study aimed to examine the Schober test and its variants, surface anatomical marker accuracy and repeatability compared to actual L1-S1 motion segmental length as measured on sagittal computed tomography, and the likely influence of patient gender and habitus on the measurements.

## 2. Methods

The study included 25 randomly selected orthopedic patients referred to our clinic in 2020.

Inclusion criteria were age over 18 years with documented anthropometric measures including BMI, height and an available recent CT scan. Patients with a history of lumbar spine surgery, fractures, infections, malignancy and spinal deformities, i.e., scoliosis or kyphosis, were excluded from the study.

**Figure 1:**
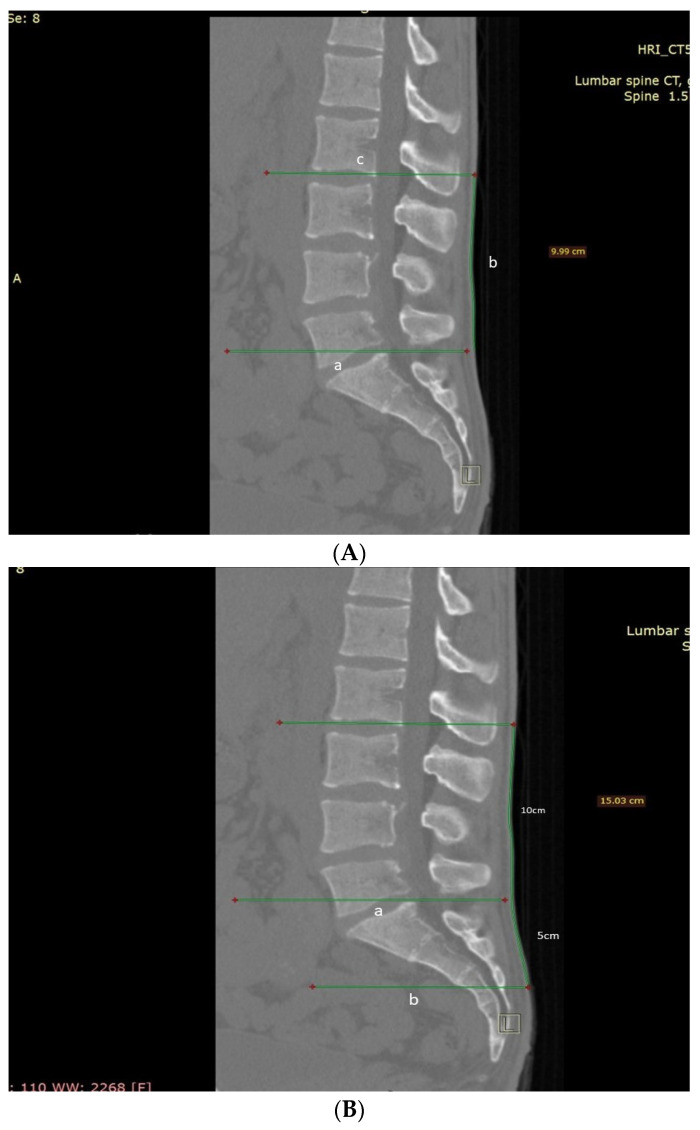
(**A**) Schober Test CT measurement. A parallel line is drawn from the posterosuperior corner of the S1 vertebra to the skin (marked as a), which is followed by a 10 cm line drawn over the skin to reach the cephalad endpoint imitating the original Schober test (marked as b). A parallel line was drawn from this point to the skeletal spine level, transecting a vertebra (upper third, middle third, or lower third) or a disc space (marked as c). Lumbar vertebrae were numbered as (L)1 to (L)5, with the upper third as 0.3, middle third as 0.6, and lower third as 0.9. A line transecting the middle third of L2 was therefore designated as 2.6. Sacral vertebras were numbered from 6 to 10 similarly. (**B**) Modified Schober Test CT measurement. It was measured similarly to the Schober test by marking a skin point 5 cm caudal and 10 cm cephalad to the lumbosacral junction (a parallel line drawn from the posterosuperior corner of the S1 vertebra to the skin) (Marked as a). Spinal endpoints were measured; the cephalad point was measured as in the Schober test. The caudal end was measured as the parallel line from the caudal 5 cm skin point to the skeletal spine level (upper third, middle third, or lower third of a vertebra or the disc space) (Marked as b).

**Figure 2:**
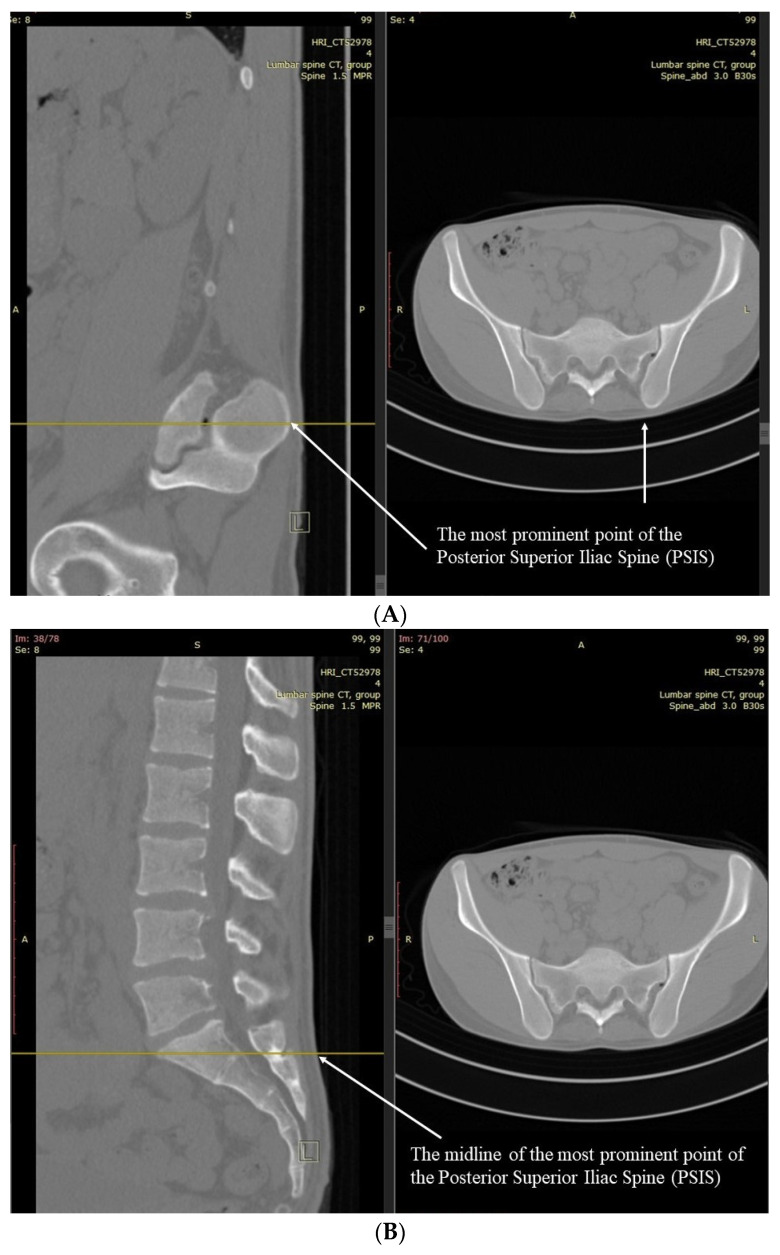

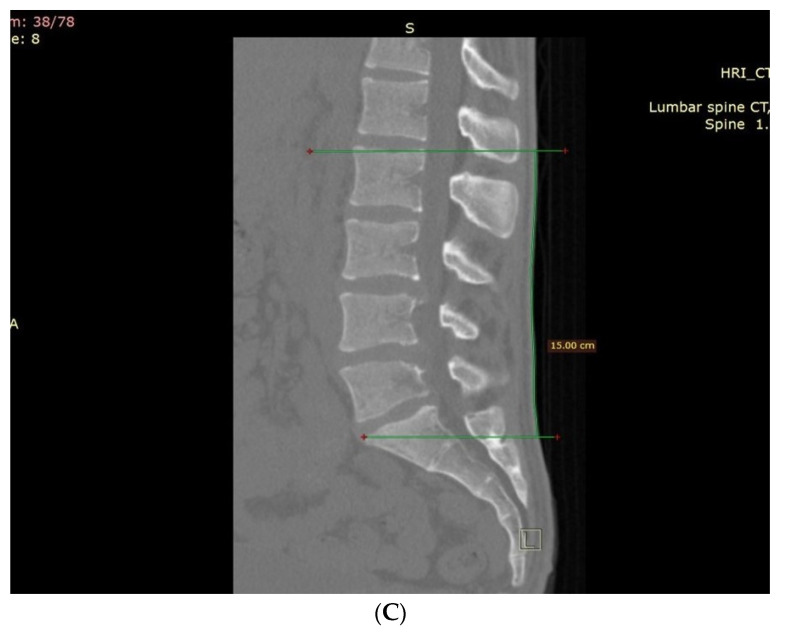
MMST Measurement. (**A**) MMST PSIS LANDMARK on CT axial–sagittal views. (**B**) MMST PSIS Qaudal starting point. MMST was measured by marking the most prominent point of the Posterior Superior Iliac Spine (PSIS), as measured by CT (Marked arrows). On that level, scrolling back to a midline sagittal plane, a parallel line was drawn to the skin surface as described previously. (**C**) MMST complete CT measurement. From that point, a 15 cm skin surface line was drawn cranially. The caudal and cephalad spinal levels were measured as described before.

**Figure 3:**
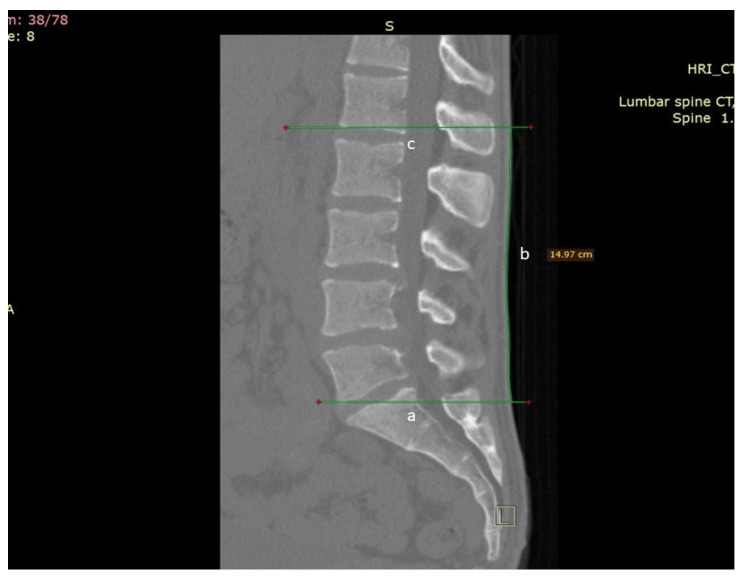
Actual lumbar (L1-S1) motion segments length measurement on CT. A line was stretched from the mid-endplate of S1 to the skin surface (marked as a), and a parallel line was extended from the mid-endplate of L1 to the skin surface (marked as c). The distance between both lines was measured on the skin surface (marked as b).

Two fellowship-trained spinal consultants assessed all 25 patients’ CT scans for the skin level of the Schober test and MST skin surface starting position and the MMST upper and lower skin levels of the measured starting position (Figure 1 and Figure 2). The same surgeons also measured the actual L1-L5 skin length (Figure 3) and the Wolfson MMST levels.

The Schober test was initially described as skin marking 10 cm cephalad to the lumbosacral junction, including L1-S1 motion segments. In our study, this was a parallel line drawn from the posterosuperior corner of the S1 vertebra to the skin, which was followed by a 10 cm line drawn over the skin to reach the cephalad endpoint imitating the original Schober test. A parallel line was drawn from this point to the skeletal spine level, transecting a vertebra (upper third, middle third, or lower third) or a disc space illustrated in Picture 1. Lumbar vertebrae were numbered as (L)1 to (L)5, with the upper third as 0.3, middle third as 0.6, and lower third as 0.9. A line transecting the middle third of L2 was therefore designated as 2.6. Sacral vertebras were numbered from 6 to 10 similarly.

MST was measured similarly to the Schober test by marking a skin point 5 cm caudal and 10 cm cephalad to the lumbosacral junction (a parallel line drawn from the posterosuperior corner of the S1 vertebra to the skin). Spinal endpoints were measured; the cephalad point was measured as in the Schober test (Figure 1B). The caudal end was measured as the parallel line from the caudal 5 cm skin point to the skeletal spine level (upper third, middle third, or lower third of a vertebra or the disc space—Figure 1B).

MMST was measured by marking the most prominent point of the Posterior Superior Iliac Spine (PSIS), as measured by CT (Figure 2A,B). On that level, scrolling back to a midline sagittal plane, a parallel line was drawn to the skin surface as described previously. From that point, a 15 cm skin surface line was drawn cranially. The caudal and cephalad spinal levels were measured as described before (Figure 2C). Actual lumbar (L1-S1) motion segments length measurement on CT was also measured. A line was stretched from the mid-endplate of S1 to the skin surface (marked as a), and a parallel line was extended from the mid-endplate of L1 to the skin surface (marked as c). The distance between both lines was measured on the skin surface (marked as b) (Figure 3).

All measurements were summed and coded for statistical analysis. R Statistical Software, version 3.5.2 (Foundation for Statistical Computing, Vienna, Austria), was used to perform statistical analyses. Correlations between each of the Schober test modifications and patients’ gender, Body Mass Index (BMI), Weight, Height, and Lumbar Lordosis were examined using Student’s *t*-tests.

The Schober test (ST), Modified Schober (MS) test, and Modified–Modified Schober (MMS) test results were compared between the two examining physicians using Pearson correlations and were found to be significantly correlated (Pearson coefficient for the total scores = 0.99, *p*-value < 0.001).

Our institute IRB committee has approved this project without the patient’s consent required from each patient.

## 3. Results

We have summarized the measurements of 25 computer tomography (CT) studies of random patients out of our spine clinic, excluding patients with prior history of spine surgery, fracture, or deformity. The cohort’s average age was 57.3 years (CI 18–84), 60% were males with an average BMI of 27.7 (CI 21–41.4), and 40% were women with an average BMI of 28.2 (CI 21–38.6) (Table 1). Men were taller (175.4 cm, CI 168–192) than women (161.2 cm, CI 151–173). Men’s lumbar lordosis was lower than women’s (48.1 vs. 56 degrees, *p* = 0.02). The Schober test, MST, MMST, and the actual skin surface length of L1-S1 motion segments were measured on each CT scan (Figure 1, Figure 2 and Figure 3).

Simulating the original Schober test on the CT scans (Figure 1) showed that this test failed to include the L1-L2 motion segment in all included patients. The Schober test ended in L2 in 17 patients (68%, eight men and nine women) and in L3 in another 8 cases (32%, seven men and one woman). None of the measurements performed encompassed all the intended lumbar motion segments, thus failing to assess the full lumbar spine motion (Table 2).

The Modified Schober test (MST), which added 5 cm caudally, did not improve the cephalad measurement: thus not improving lumbar spinal motion measurement precision but merely overcoming the difficulty in identifying the L5-S1 junction. The MST maintains all the disadvantages of the Schober test, missing the intended motion segments of L1-S1 in all cases.

When measuring the CT based Modified–Modified Schober Test (MMST), using a 15 cm distance cranial to the PSIS landmarks, 19 patients had a full L1-S1 motion segment assessment (76% accuracy, 73.3% in men and 80% in women). In contrast, in six cases (four men and two women), the L1-L2 motion segment was missed altogether. MMST was inaccurate in 24% of the measurements, better accounting for potential L1-S1 motion than the Schober test and MST, but still having a substantial error.

Based on those results, we have devised another modification to the MMST by adding one cm to the MMST measurement, thus measuring 16 cm cephalad to the PSIS. The simulated 16 cm modification (Wolfson MMST (WMST)) improved the measurement significantly. WMST measurement included the L1-S1 motion segments, as initially intended, to clinically assess the full extent of the lumbar spine motion in all of the cases. We had one case of a 170 cm male in which an additional motion segment was included (T12-L1).

**Figure 4:**
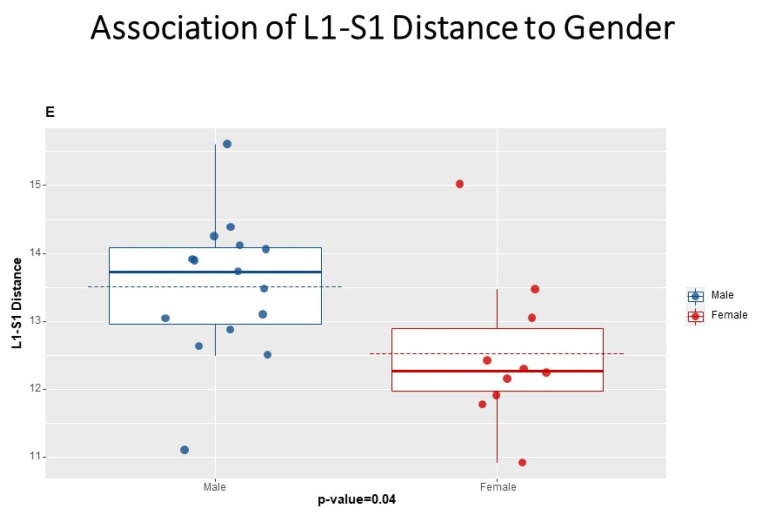
Association of L1-S1 Distance to Gender.

We further studied the influence of morphometric parameters; Height, Weight, Body Mass Index (BMI), and gender over the Schober test, MST, MMST, and L1-S1 skin distance. When studying the L1-S1 actual skin distance, we found a significant association with weight (*p* = 0.047), height (*p* = 0.015), and gender (*p* = 0.04). Lumbar lordosis and BMI showed a similar tendency but did reach statistical significance (*p* = 0.147 and *p* = 0.322, respectively). Gender was found to influence all of the studied exams (P ranges from 0.02 to 0.05) except for the MMST (*p* = 0.6), with males being higher than females and with longer L1-S1 skin length (Figure 4).

## 4. Discussion

Health professionals routinely examine the lumbar range of motion, i.e., physicians of all fields and physiotherapists. Lumbar ROM is an essential clinical tool for assessing disease progression (such as ankylosing spondylitis or degenerative spine disease), patient recovery (following surgery or injury) and quantifying disability (such as in compensation claims) [6,34,35,36,37,38].

The first commonly used tool for measuring lumbar ROM is the Schober test [24]. Its limitations include difficulty identifying the L5-S1 junction at the skin level and the uncertainty of whether the 10 cm interval encompasses the L1-S1 motion segments. Our study showed that with an accurate CT scan-based starting point, the 10 cm interval fails to include 68% of the L1-L2 motion segments and 32% of the L2-3 motion segment. Based on these results, the Schober test is highly inaccurate and should be abandoned entirely.

The Modified Schober test was supposed to overcome the Schober test’s starting point ambiguity without changing the 10 cm examination span [25]. This modification inherits the inaccuracy of the Schober Test’s L5-S1 skin starting point and adds caudal length to measure non-mobile sacral segments without improving the measurements of the mobile lumbar segments. Therefore, this modification is inaccurate, as the Schober test and should be avoided as well.

The last modification described was the Modified–Modified Schober Test [22], bypassing the L5-S1 junction and reliance on the PSIS bony landmark as an identifiable starting point with a 15 cm interval that was supposed to include the entire L1-S1 motion segments. The MMST gained wide acceptance among health professionals due to its improved accuracy [21,33]. Our measurements validated this by proving significant improvement of the L1-S1 measurement from 32% accuracy of the Schober/MST to 76% using the MMST. The MMST still failed to include the L1-2 segment in 24% of patients, remaining a suboptimal assessment of the L1-S1 motion segments.

With the MMST advantage of PSIS bony landmarks being a better anatomical anchor than the L5-S1 junction, we re-examined the CT scans with a 16 cm interval length. We found that this modification, Wolfson Modified Schober Test (WMST), improved the accuracy of measuring L1-S1 to 96% with only one case, which also included the T12-L1 motion segment. The significance of including the T12-L1 motion segment should have less impact on lumbar flexion–extension motion than disregarding the L1-2 motion segment, as seen in the MMST [39].

This study used supine lumbar spine CT scans for measurement, raising the question of positioning effect on lumbar lordosis and Schober measurement techniques. At the same time, some reports have described significant positional changes in lordosis and scoliosis for patients with spinal deformities [40,41]. Others showed a minimal change, of about 3 to 6 degrees, mainly in the healthy adult population with normal spinal alignment [42,43,44,45,46]. Since our study’s cohort did not include deformities, the results are probably unbiased by position.

All Schober test measurement techniques previously described did not consider the subject’s BMI as a possible bias, and no BMI measurement modifiers were suggested. Our cohort was heterogenous in regard to BMI [21,22,23,24,25,26,27,28,29,30,31,32,33,34,35,36,37,38,39,40,41], but still, the Wolfson Modified Schober Test (WMST) was more accurate than the currently described tests in various BMIs.

This study’s main limitations include a relatively small patient sample and the fact that CT-based measurements were performed on supine patients instead of upright, which might marginally affect measurements due to altered lordosis and disc loading. However, the results proved to be statistically significant.

In conclusion, the Schober test and all of its modifications so far are lacking in L1-S1 motion segment inclusion, and therefore, they are prone to underestimation of lumbar spine motion. This study shows that the WMST is much more accurate than previous modifications, thus better evaluating lumbar spine motion. Precise assessment of the lumbar range of motion is essential for disability measurement, since many health systems worldwide use this as the main lumbar disability parameter. Further studies to measure the average ROM data of a healthy population are required before the wide usage of the WMST.

## Figures and Tables

**Table 1:** Cohort demographics (*n* = 25).

Sex (M:F,%)	Age	Height	Weight	BMI
60:40	57.3 (CI 18–84)	169.8 (CI 145–192)	80.3 (CI 51–134)	27.7 (CI 21–41.4)

**Table 2:** Frequency and vertebral level transected by each measurement method *.

**Schober Test**								
**Av. Level**	2.45	2.6	2.75	2.9	3.1	3.3	3.6	3.75
**Frequency**	1	4	4	8	3	3	1	1
**%**	0.04	0.16	0.16	0.32	0.12	0.12	0.04	0.04
**MS Caudal Level**								
**Av. Level**	8.1	8.3	8.4	8.45	8.6	8.75	8.9	9.1
**Frequency**	1	1	1	5	4	6	3	1
**%**	0.04	0.04	0.04	0.2	0.16	0.24	0.12	0.04
**MMS Cephalad Level**								
**Av. Level**	1.1	1.45	1.6	1.75	1.9	2.1	2.25	2.45
**Frequency**	2	2	3	4	7	4	1	1
**%**	0.08	0.08	0.12	0.16	0.28	0.16	0.04	0.04

* Lumbar vertebrae were numbered as 1 to 5, with the upper third as 0.3, middle third as 0.6, and lower third as 0.9. A line transecting the middle third of L2 was therefore designated as 2.6. Sacral vertebras were numbered from 6 to 10 similarly. All values were an average value of the two measures (independent measure by each consultant).

## Data Availability

Not applicable.

## Abbreviations

Schober test (ST), Modified Schober test (MST), Modified–Modified Schober Test (MMST), computed tomography (CT), Wolfson MMST (WMST).
